# A Near Gap‐Free Haplotype‐Resolved Genome Assembly of *Zoysia japonica* Uncovers Intra‐Subgenomic Gene Expression and Regulatory Variation

**DOI:** 10.1111/pbi.70634

**Published:** 2026-03-09

**Authors:** Sae Hyun Lee, Preethi Purushotham, Ambika Chandra, Murukarthick Jayakodi

**Affiliations:** ^1^ Texas A&M AgriLife Research and Extension Center Dallas Texas USA; ^2^ Department of Soil and Crop Sciences Texas A&M University College Station Texas USA

Zoysiagrass (*Zoysia* spp. Willd.) is a perennial warm‐season turfgrass widely used in lawns, sports fields, and urban landscapes across the southern United States and East Asia (Patton et al. 2017). Species in this genus are allotetraploids (2n = 4× = 40) with compact genomes (~300–400 Mb) and exhibit extensive variation in tolerance to multiple abiotic stresses, making them important targets for breeding and stress biology research (Tanaka et al. 2016). *Zoysia japonica* Steud. and *Z. matrella* (L.) Merr. are the primary species used in breeding programs (Chandra et al. 2017). However, high heterozygosity resulting from outcrossing has limited genome assembly quality, often yielding haplotype‐collapsed references that obscure allelic and regulatory variation and restrict the development of phased markers for genetic mapping and breeding. Here, we report a near gap‐free, fully chromosome‐phased genome assembly of the *Z. japonica* cultivar ‘Palisades’, a widely used coarse‐textured zoysiagrass in the southern United States.

First, a fully phased *de novo* contig assembly was generated using PacBio HiFi reads and Hi‐C data (Table S1) with hifiasm (Cheng et al. 2021), and subsequently scaffolded to the chromosome‐scale using the allele‐aware HapHiC (Zeng et al. 2024). Chromosome numbers were assigned based on alignment with the genetic map (Wang et al. 2015). The high quality of the assembly was supported by clear diagonal Hi‐C contact matrices (Figure S1) and strong concordance with the genetic map (Figure S2). The final assembly comprised 20 chromosome pairs, totaling 317.2 Mb for haplotype 1 (hap‐1) and 317.0 Mb for haplotype 2 (hap‐2) (Table S2, Figure 1A). All chromosomes were gapless, except for five chromosomes in hap‐1 and one in hap‐2 (Table S2). Telomeric repeats were detected at both ends of all chromosomes (Figure S3), indicating telomere‐to‐telomere continuity in the genome. Gene space assessment by BUSCO identified 98.3% of conserved Poales orthologs genes, and assembly evaluation by Merqury estimated 99% completeness with a consensus quality of 76.5 (Table S3), confirming the high accuracy of the assembly. Because the progenitors of *Z. japonica* remain unidentified, we attempted subgenome assignment using *k*‐mer profiling by SubPhaser (Jia et al. 2022) and phylogenetic approaches; however, both methods were inconclusive, indicating the requirement for genomic information from at least one diploid progenitor. Hap‐1 and hap‐2 showed overall collinearity with the previously published haplotype‐collapsed *Z. japonica* cultivar ‘Compadre’ assembly (Figure S4) (Shen et al. 2025). However, each Palisades haplotype exhibited a distinct set of structural sequence differences relative to Compadre (Table S4), consistent intraspecific variation. Furthermore, comparison of raw HiFi read alignments at regions where structural variants (SVs) were identified between hap‐1 and hap‐2 in Palisades revealed chimeric alignments in the corresponding regions of the Compadre assembly (Figure S5). These patterns indicate mosaic allelic representation in the haplotype‐collapsed assembly and underscore the necessity of phased genome assemblies. Consistent with previous studies (Tanaka et al. 2016; Wang et al. 2015), both haplotypes also exhibited strong collinearity with rice ( *Oryza sativa* IRGSP‐1.0) and sorghum ( *Sorghum bicolor* NCBIv3) (Figure S6), highlighting the utility of *Z. japonica* for cross‐species comparative genomics and translational research.

**FIGURE 1:**
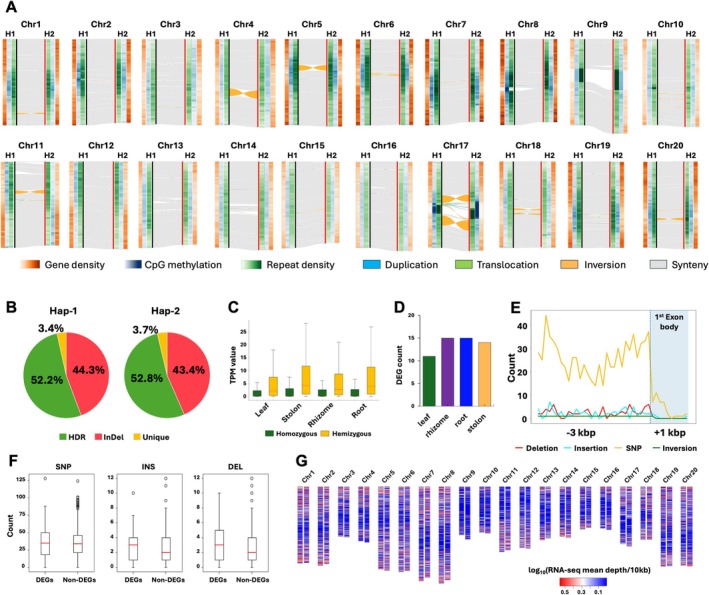
Phased homologous chromosomes and regulatory landscapes in *Z. japonica* . (A) Whole‐genome collinearity between haplotypes showing gene density, CpG methylation, repeat density, and major structural rearrangements. (B) Hemizygous genes overlapping highly diverged regions (HDRs) and insertions/deletions (InDels) identified by SyRI; genes present in only one haplotype are classified as unique. (C) Transcript abundance (TPM) of homozygous and hemizygous genes across four tissues. (D–F) Cis‐regulatory differentially expressed genes and sequence variants within 3‐kb upstream regions. (G) Expression differences between hap‐1 and hap‐2.

Annotation of repetitive sequences accounted for approximately 50% of each haplotype (158 Mb in hap‐1 and 156 Mb in hap‐2) (Table S5). Gene prediction, integrating short‐ and long‐read transcriptome data (Table S6) and protein homology, after our rigorous manual curation (Figure S7), identified 40 106 and 40 223 protein‐coding genes in hap‐1 and hap‐2, respectively (Table S7), with 96% BUSCO completeness. Comparison of hap‐1 and hap‐2 using SyRI (Goel et al. 2019) revealed extensive collinearity (~250 Mb per haplotype) and substantial haplotype‐specific sequences (142 Mb in hap‐1 and 141 Mb in hap‐2) (Figure 1A). Notably, we identified approximately 2.9 million SNPs and numerous structural variants (SVs), including 188 877 insertions, 196 125 deletions, 46 inversions, and 52 translocations (Table S8, Figure 1A). Most SVs were < 10 kb, with only 4 inversions exceeding 1 Mb (Table S8). PCR validation of five randomly selected SVs confirmed their authenticity and precise breakpoints (Figure S8, Table S9), underlining the accuracy of the assembly and suitability of developing phased DNA markers. A substantial number of haplotype‐specific hemizygous genes were detected between hap‐1 and hap‐2. In total, 4863 (12%) and 4519 (11%) genes were specific to hap‐1 and hap‐2, respectively (Figure 1B). Of the hap‐1–specific genes, 44.3% (2154) were located in hap‐1–specific insertions, 52.2% (2539) in highly divergent regions (HDRs) identified by SyRI, and 3.4% (169) in haplotype‐unique sequences absent from the alternate haplotype. A similar pattern was observed in hap‐2, with 1962 (43.4%) genes positioned within insertion regions, 2389 (52.8%) within HDRs, and 167 (3.7%) within haplotype‐unique regions. These results indicate that hemizygous genes arose from SV events between hap‐1 and hap‐2, as well as from divergent and unique sequences likely inherited from the progenitors of each subgenome. Importantly, these haplotype‐specific hemizygous genes are not merely annotation outcomes or pseudogenes; they exhibit relatively higher expression levels compared with homozygous diploid genes (Figure 1C). Furthermore, to explore the *cis*‐regulatory landscape between hap‐1 and hap‐2, we identified 539 one‐to‐one identical homologous genes with 100% coding sequence identity and examined their expression across four tissues (leaf, stolon, rhizome, and root). Among these, 29 genes showed significant differential expression (DE) between haplotypes (Figure 1D). High numbers of sequence variants, including SNPs and SVs, were identified in the 3‐kb upstream promoter regions of these DE genes (Figure 1E) compared to non‐DE genes (*n* = 510) (Figure 1F), suggesting expression difference between haplotypes likely due to *cis*‐regulation. Collectively, these findings reveal that SVs, HDRs, haplotype‐unique sequences, and *cis*‐regulatory divergence underlie extensive transcriptional differences between haplotypes (Figure 1G, Figure S9). Our haplotype‐resolved *Z. japonica* genome enables dissection of intra‐subgenomic variation and its regulatory effects. Haplotype‐specific transcription driven by structural and *cis*‐regulatory divergence accentuates the value of phased genomes for phenotype prediction and precision breeding in heterozygous polyploids. The extensive haplotype‐specific variation identified here also provides a foundation for gene discovery and targeted *Zoysia* improvement.

## Author Contributions

M.J. and A.C. conceived the study and designed the experiments. P.P. performed DNA and RNA extractions. S.H.L. conducted genome assembly, annotation, and bioinformatic analyses. M.J., S.H.L., and P.P. wrote the manuscript. All authors reviewed and approved the final version.

## Conflicts of Interest

The authors declare no conflicts of interest.

## Supporting information


**Figure S1:** Interchromosomal Hi‐C contact matrix. (A) Haplotype 1. (B) Haplotype 2.

**Data S1:** pbi70634‐sup‐0002‐Supinfo.docx.

**Figure S2:** Concordance between the genetic and physical maps. The x‐axis represents physical positions along the genome, and the y‐axis indicates genetic distances in centimorgans (cM). (A) Haplotype 1. (B) Haplotype 2.

**Figure S3:** Genome‐wide distribution of the telomeric repeat TTTAGGG. The x‐axis represents physical positions, and the y‐axis indicates the frequency of repeat sequences. (A) Haplotype 1. (B) Haplotype 2.

**Figure S4:** Collinearity analysis between previously reported *Compadre* (Shen et al. 2025) genome and the haplotype‐resolved *Palisade* genome generated in this study.

**Figure S5:** Candidate structural variant loci identified at marker‐developed regions in the haplotype‐collapsed assembly using long‐read mapping. Genomic regions corresponding to the SV68_INS primer are shown, from top to bottom, for the Compadre genome, Palisades Hap‐1 genome, and Palisades Hap‐2 genome. The size of each region is indicated above the corresponding track. Mapped reads are shown in light grey (SRR26800375). Small mis‐matches (≤ 50 bp) are highlighted in purple. Large mis‐matches (> 50 bp) are shown in dark grey.

**Figure S6:** Collinearity analysis between *Sorghum bicolor* and *Oryza sativa* . (A) *Sorghum bicolor* . (B) *Oryza sativa* .

**Figure S7:** Representative cases of gene annotation model curation. Blue boxes indicate curated models, and yellow boxes represent models prior to curation. MAS‐seq alignments are shown below the models. (A) An incorrectly split gene model. (B) An incorrectly merged gene model. (C) A gene model without supporting RNA evidence.

**Figure S8:** Validation of SV markers. PCR products were resolved on a 2% agarose gel. PCR gel image of randomly selected five variants between hap‐1 and hap‐2 with ladder.

**Figure S9:** Genome‐wide heatmap of RNA expression profiles in haplotype 1 and haplotype 2. From top to bottom, rows correspond to rhizome, stolon, and root.

**Table S1:** Summary of raw sequencing data.
**Table S2:** Comparison of assembly statistics between hap‐1 and hap‐2 and previous assemblies.
**Table S3:** Assembly completeness and consensus quality assessment.
**Table S4:** Structural variants identified in comparison with the previously published genome.
**Table S5:** Summary of repeat annotation.
**Table S6:** Summary of transcriptome sequencing data used in gene annotation.
**Table S7:** Gene annotation results.
**Table S8:** Structural variants counts and size distributions between haplotype 1 and haplotype 2.
**Table S9:** Information of the haplotype specific primers.
**Table S10:** Raw data resources utilised in the gene annotation.

## Data Availability

Genomic data supporting the findings of this study are available in the NCBI database (https://www.ncbi.nlm.nih.gov/sra) under the BioProject accession PRJNA1314844. The genome and annotation files are available in the FigShare database (https://doi.org/10.6084/m9.figshare.30131875).
