# Supplementary figures and images for: A Near Gap‐Free Haplotype‐Resolved Genome Assembly of Zoysia japonica Uncovers Intra‐Subgenomic Gene Expression and Regulatory Variation
Source: Plant Biotechnol J. 2026 Mar 9;24(6):4136–8. doi: 10.1111/pbi.70634 (PMC13205874; doi:10.1111/pbi.70634)

**A**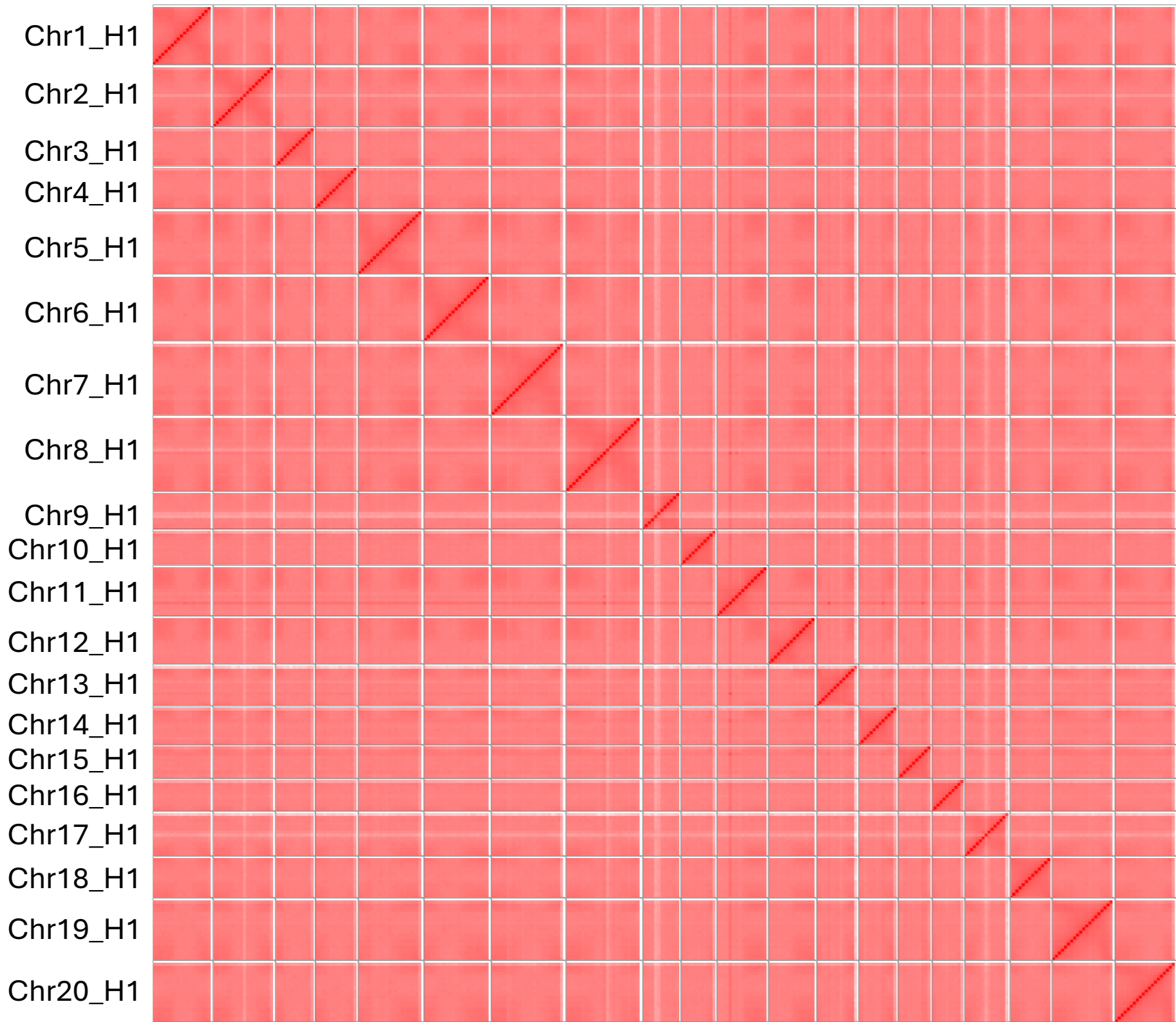**B**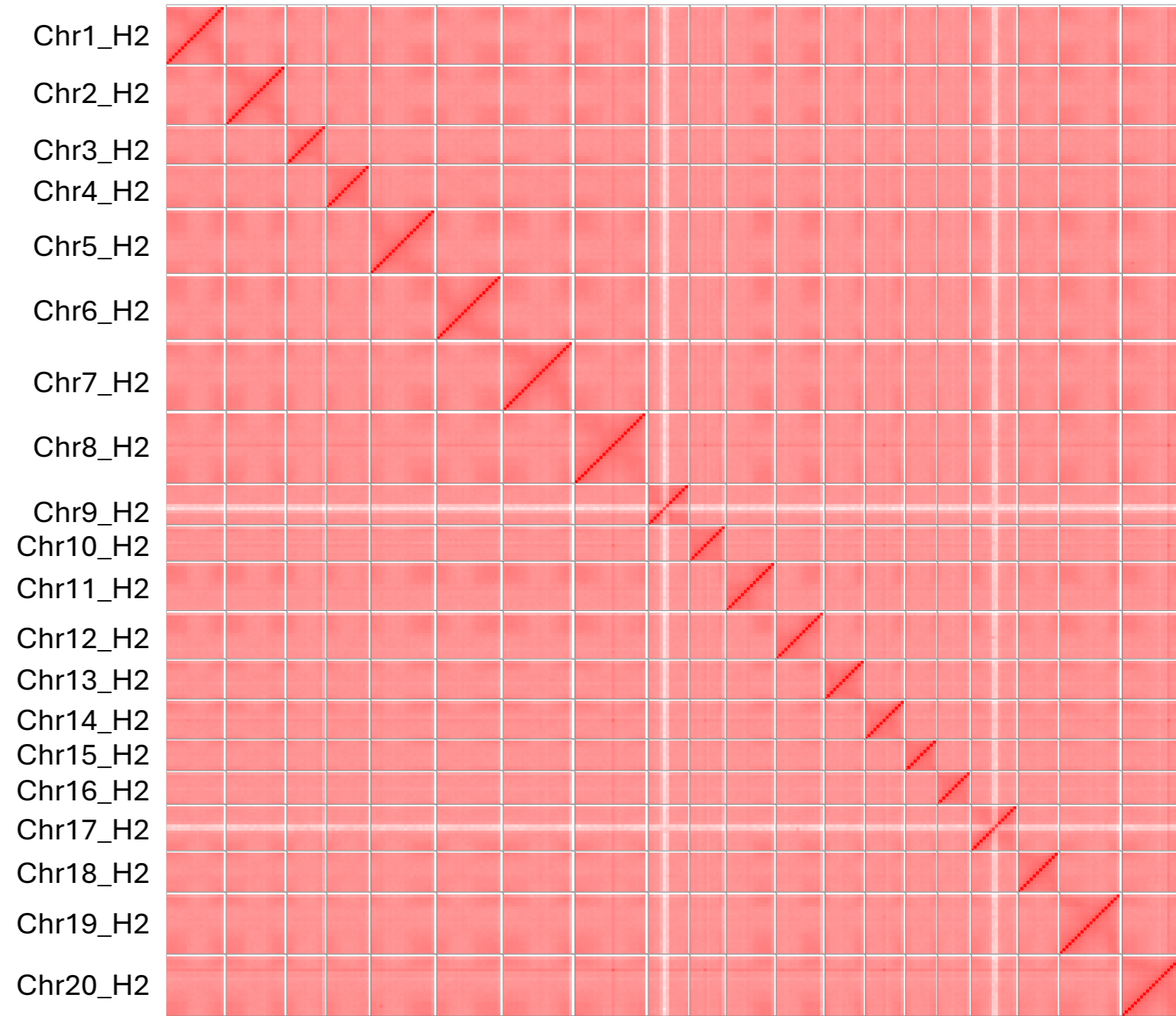

Supplement: Supplementary file 1 — Figure S1: Interchromosomal Hi‐C contact matrix. (A) Haplotype 1. (B) Haplotype 2. [file PBI-24-4136-s006.pdf]

A

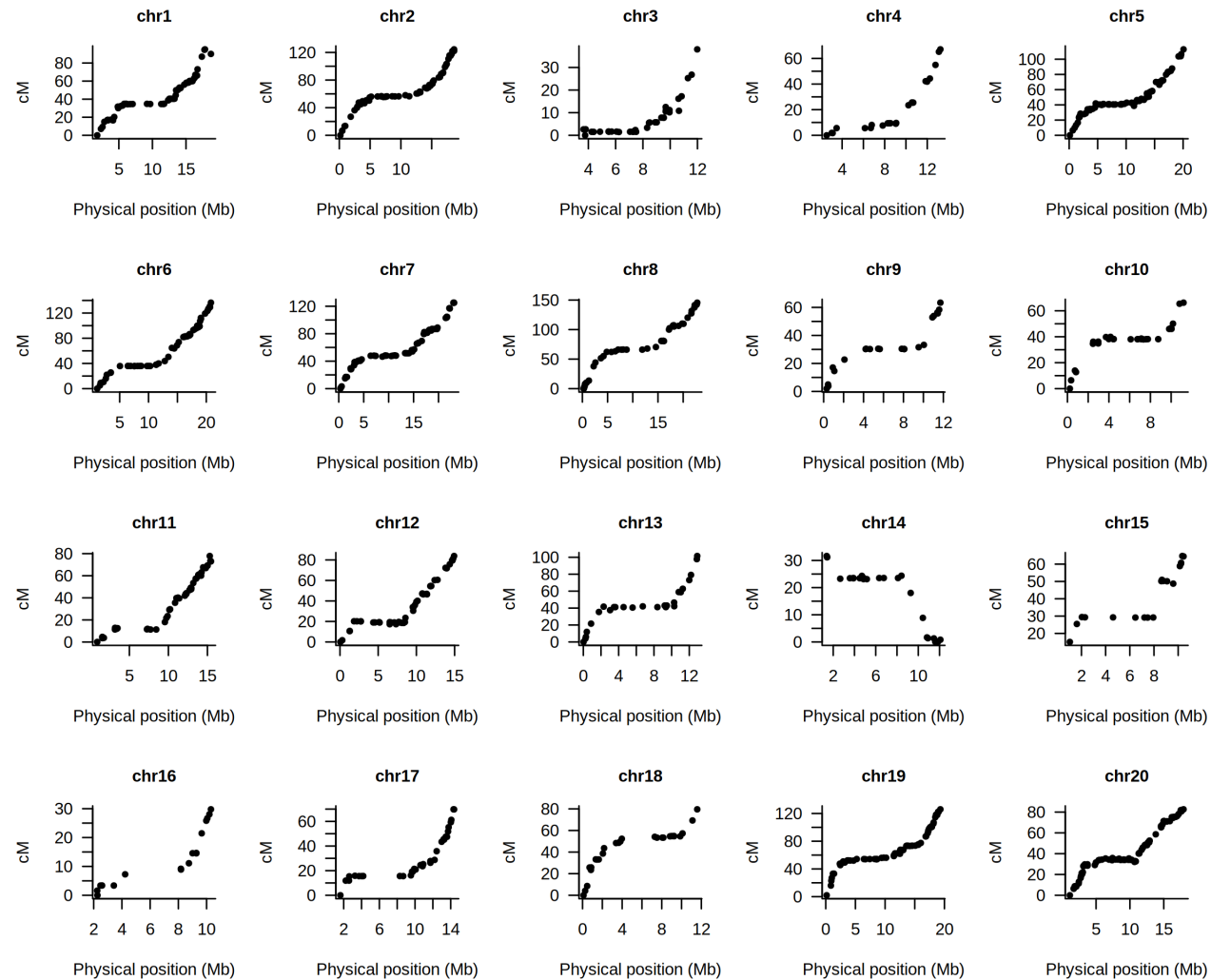

B

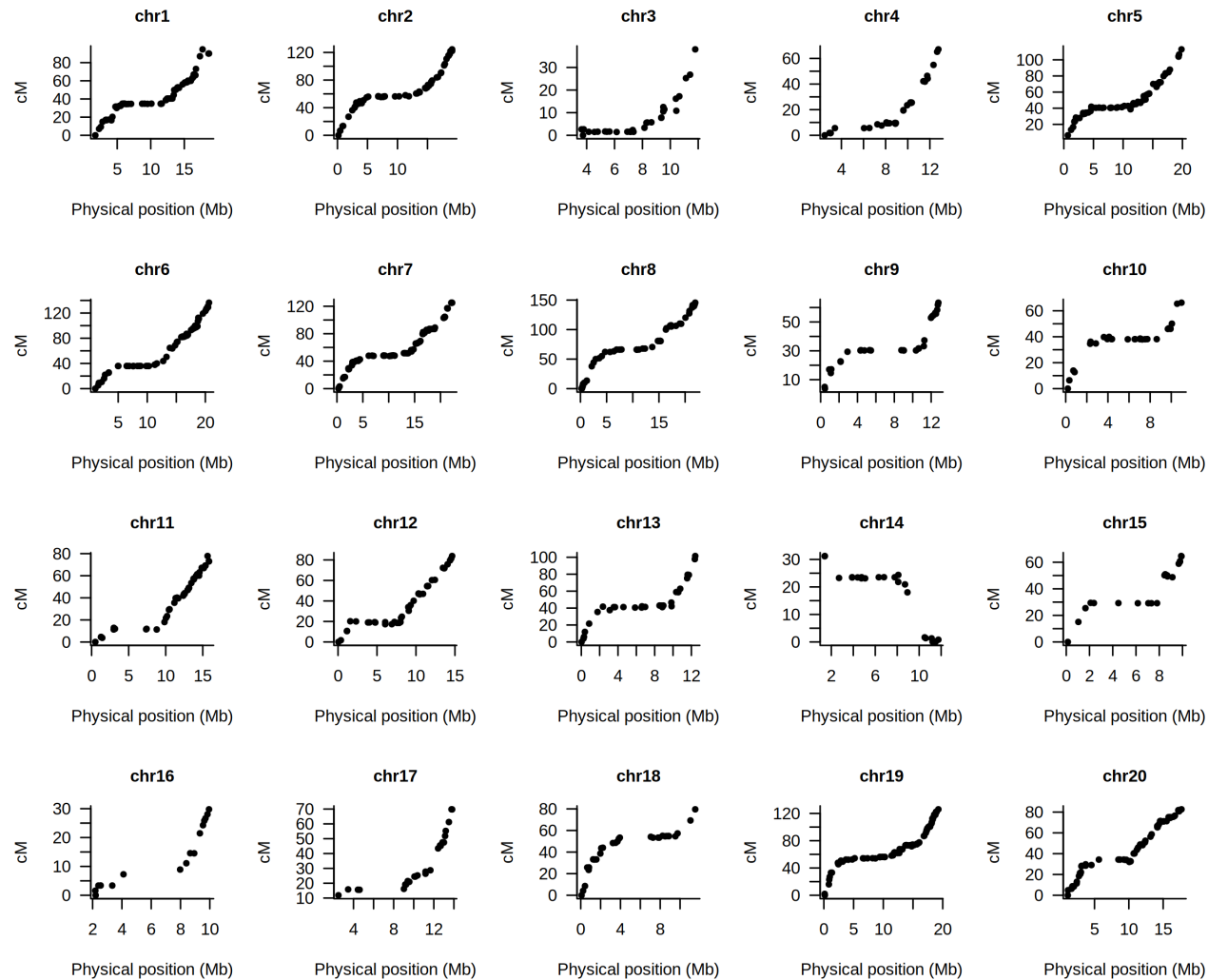

Supplement: Supplementary file 3 — Figure S2: Concordance between the genetic and physical maps. The x‐axis represents physical positions along the genome, and the y‐axis indicates genetic distances in centimorgans (cM). (A) Haplotype 1. (B) Haplotype 2. [file PBI-24-4136-s008.pdf]

A

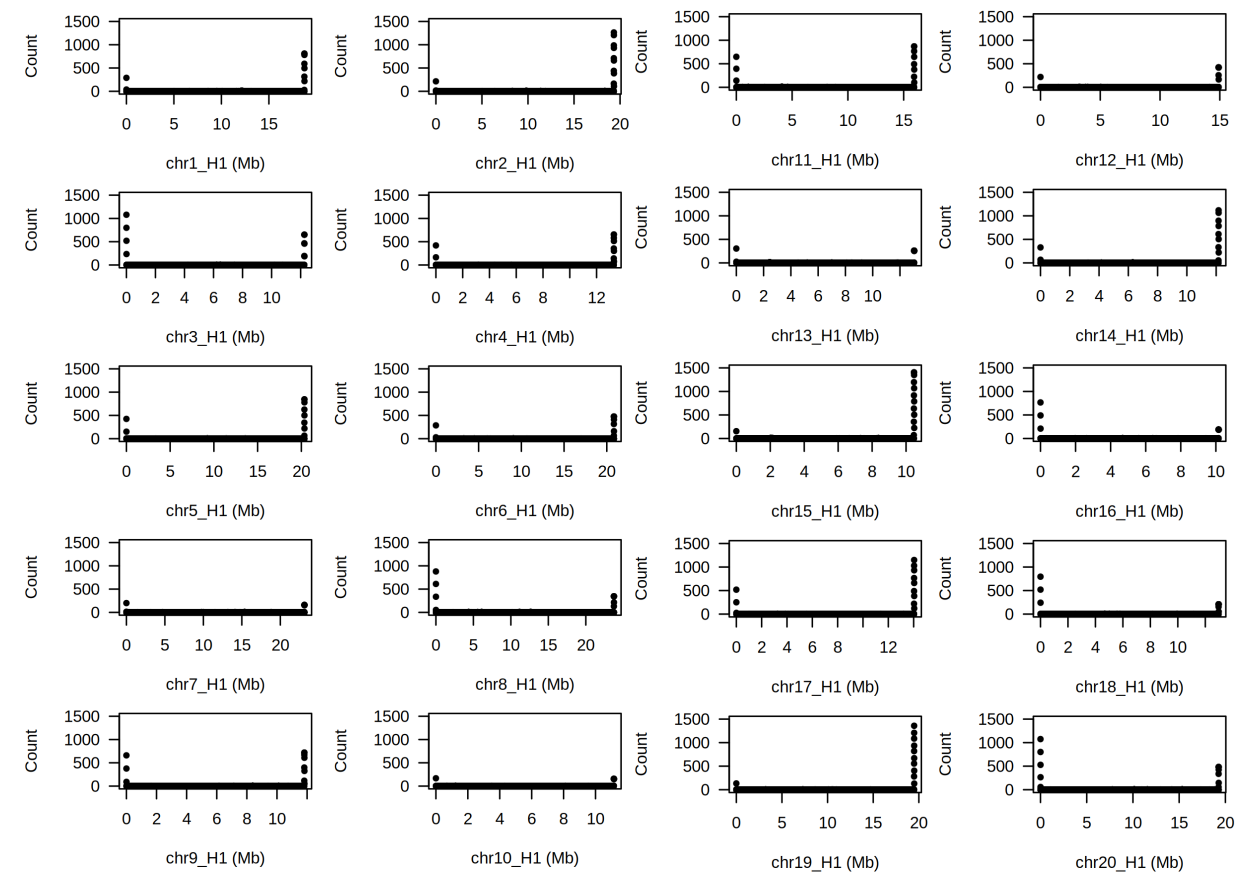

B

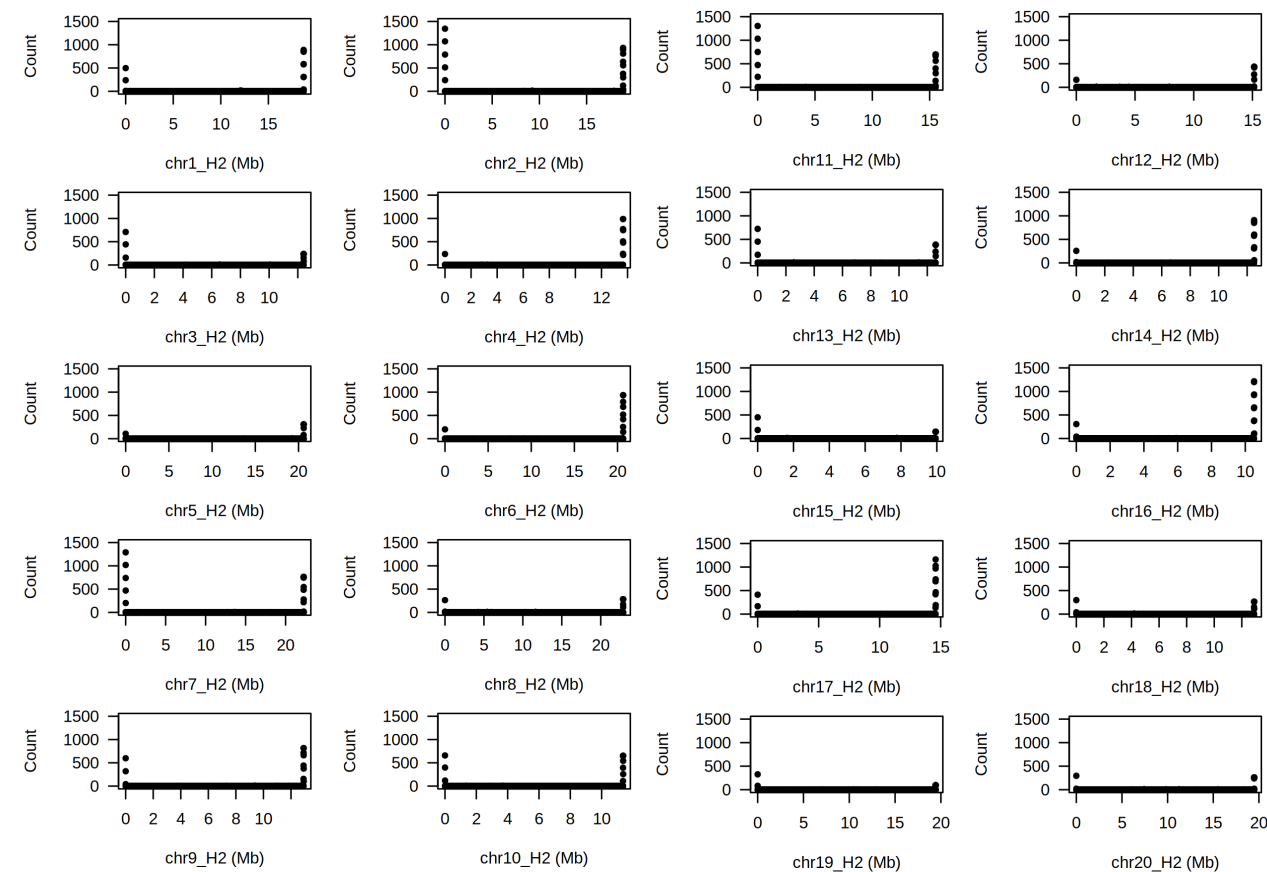

Supplement: Supplementary file 4 — Figure S3: Genome‐wide distribution of the telomeric repeat TTTAGGG. The x‐axis represents physical positions, and the y‐axis indicates the frequency of repeat sequences. (A) Haplotype 1. (B) Haplotype 2. [file PBI-24-4136-s011.pdf]

Compadre

Palisade

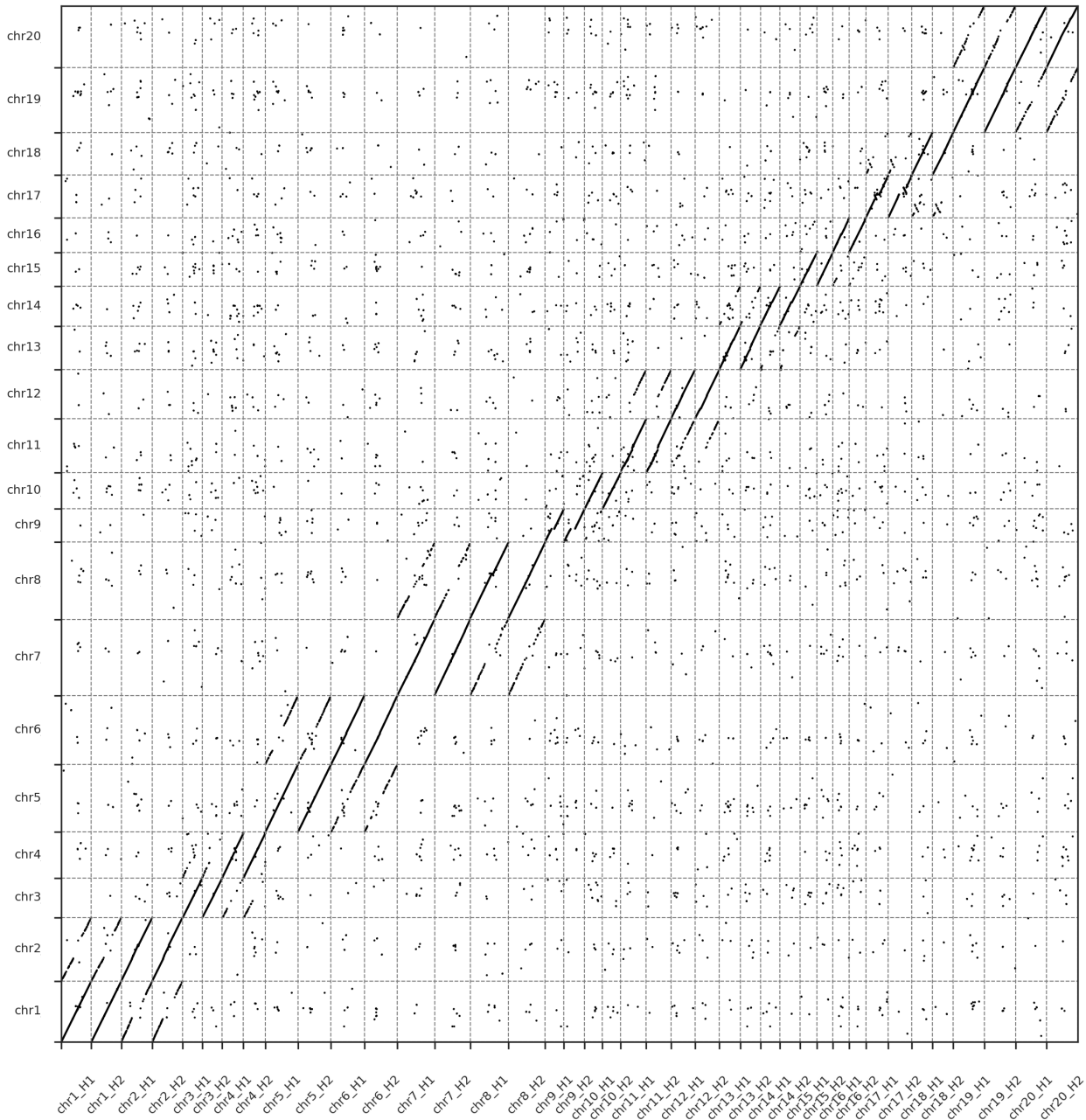

Supplement: Supplementary file 5 — Figure S4: Collinearity analysis between previously reported Compadre (Shen et al. 2025) genome and the haplotype‐resolved Palisade genome generated in this study. [file PBI-24-4136-s010.pdf]

# Compadre

6.68Kbp

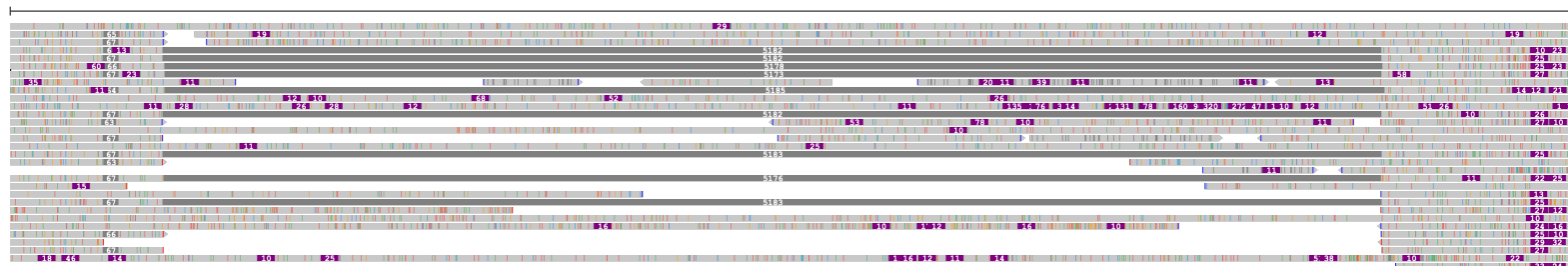

# Palisade Hap-1

6.3Kbp

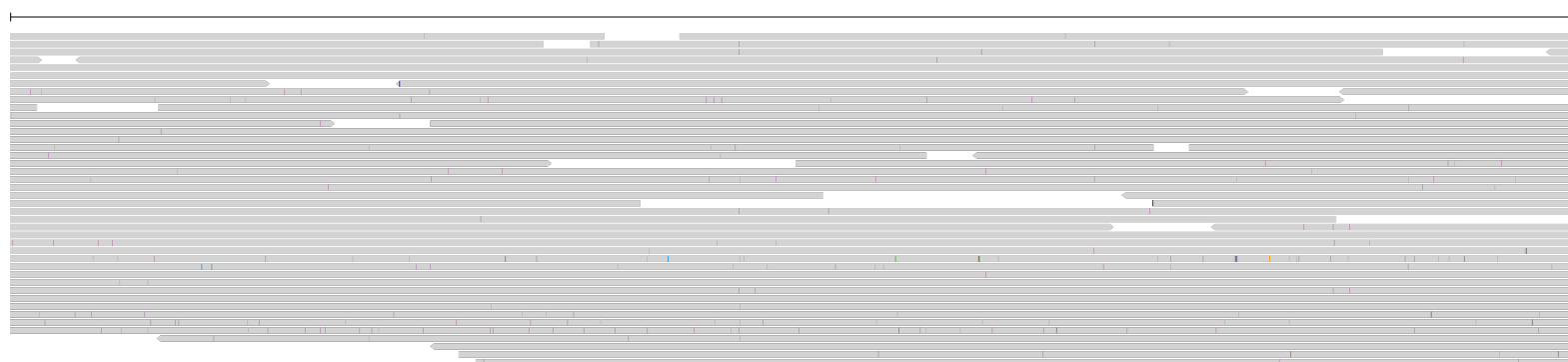

# Palisade Hap-2

6.44Kbp

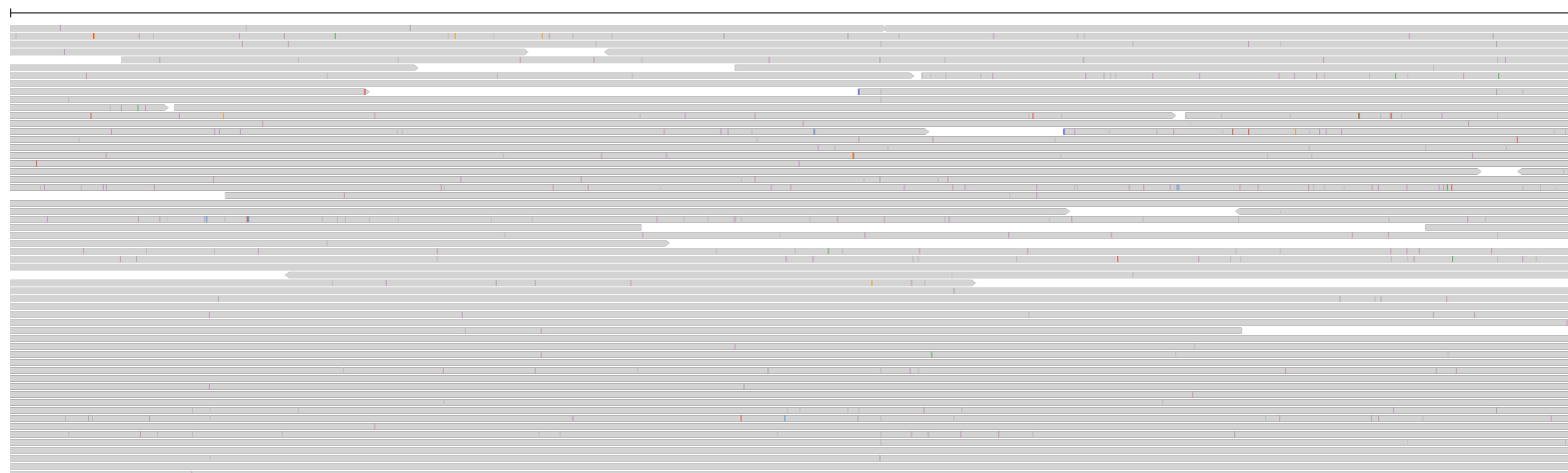

Supplement: Supplementary file 6 — Figure S5: Candidate structural variant loci identified at marker‐developed regions in the haplotype‐collapsed assembly using long‐read mapping. Genomic regions corresponding to the SV68_INS primer are shown, from top to bottom, for the Compadre genome, Palisades Hap‐1 genome, and Palisades Hap‐2 genome. The size of each region is indicated above the corresponding track. Mapped reads are shown in light grey (SRR26800375). Small mis‐matches (≤ 50 bp) are highlighted in purple. Large mis‐matches (> 50 bp) are shown in dark grey. [file PBI-24-4136-s009.pdf]

A

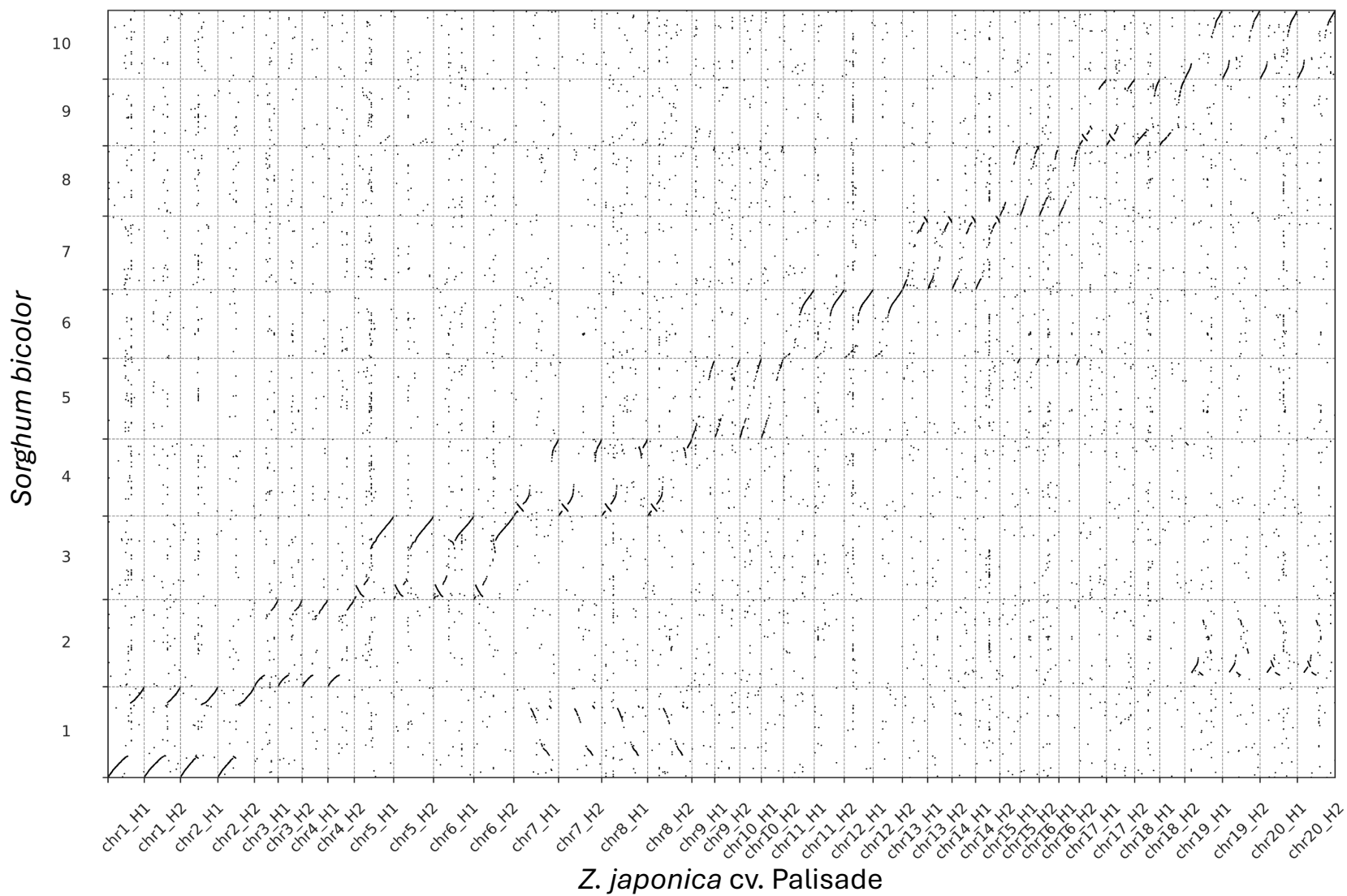

B

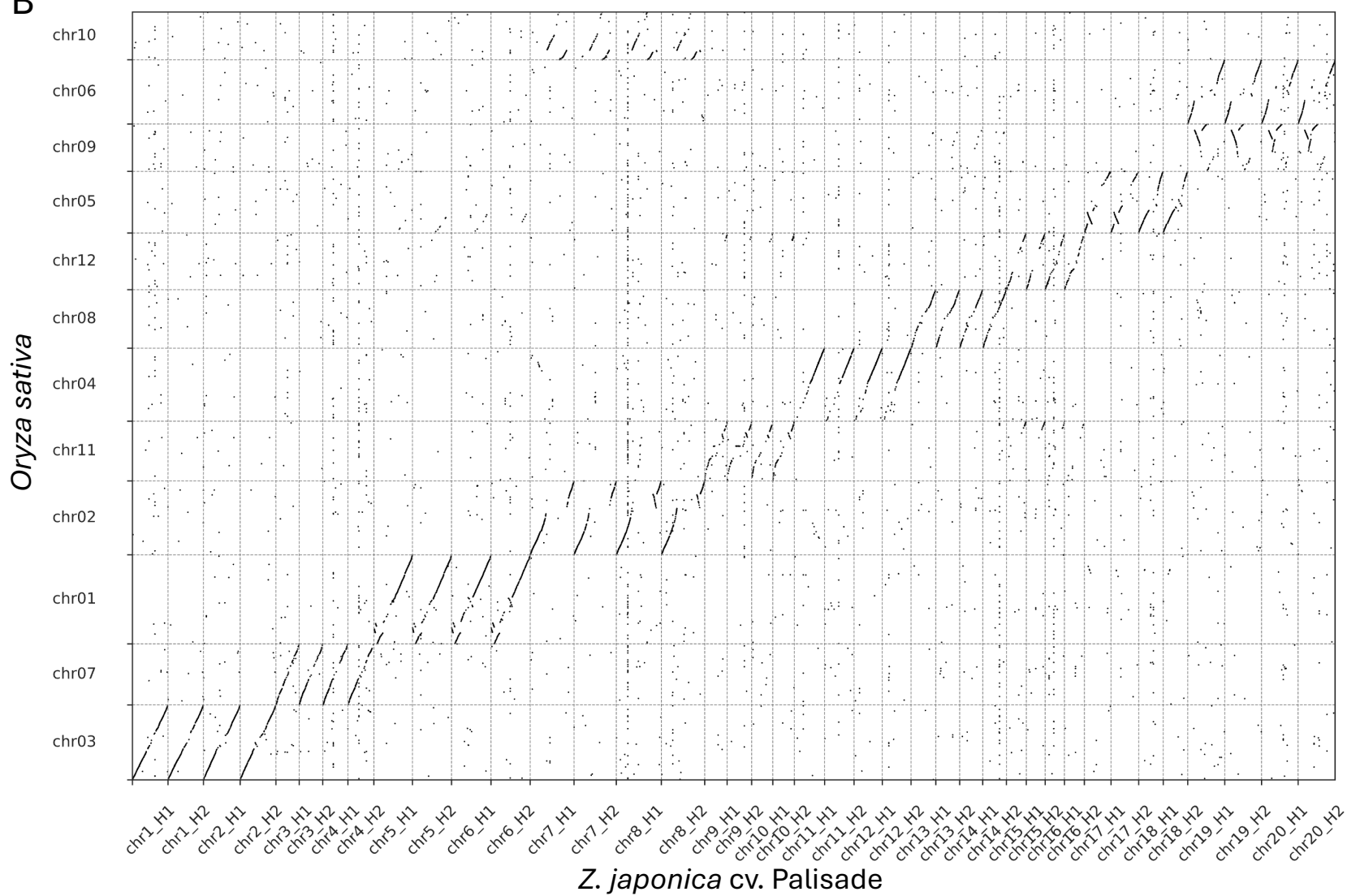

Supplement: Supplementary file 7 — Figure S6: Collinearity analysis between Sorghum bicolor and Oryza sativa . (A) Sorghum bicolor . (B) Oryza sativa . [file PBI-24-4136-s007.pdf]

A

Incorrectly split  
gene model

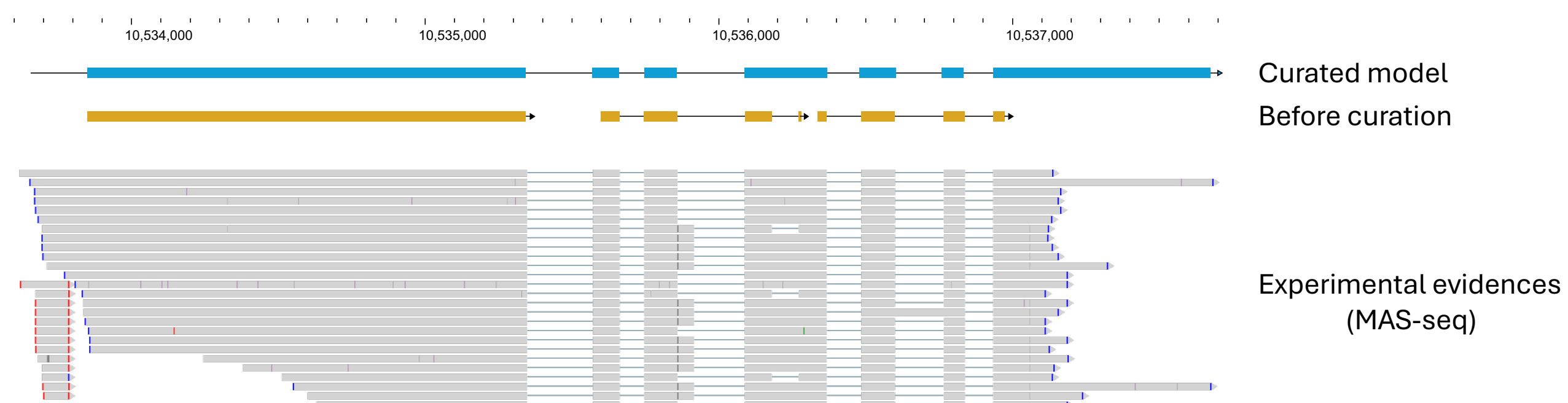

B

Incorrectly merged  
gene model

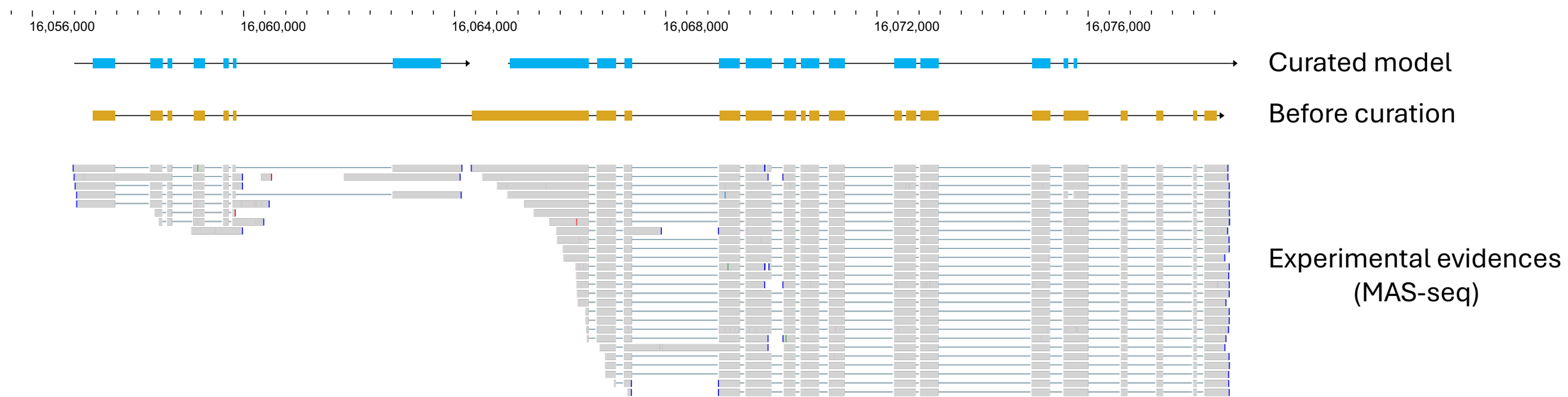

C

Missing RNA-seq  
evidence

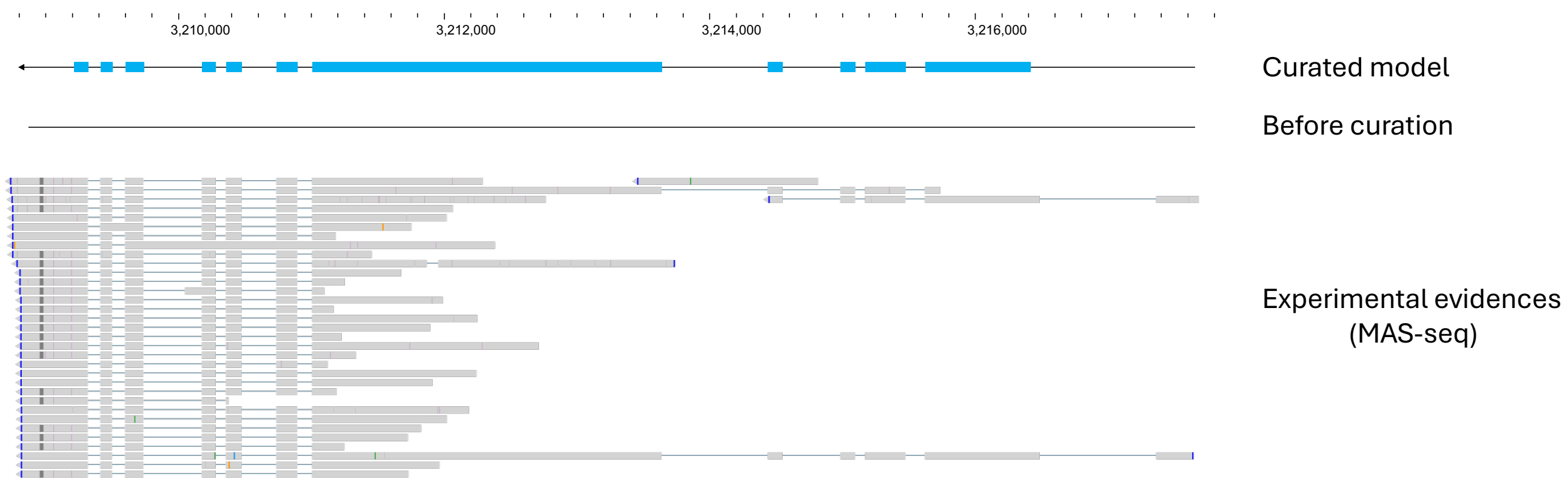

Supplement: Supplementary file 8 — Figure S7: Representative cases of gene annotation model curation. Blue boxes indicate curated models, and yellow boxes represent models prior to curation. MAS‐seq alignments are shown below the models. (A) An incorrectly split gene model. (B) An incorrectly merged gene model. (C) A gene model without supporting RNA evidence. [file PBI-24-4136-s002.pdf]

SV27

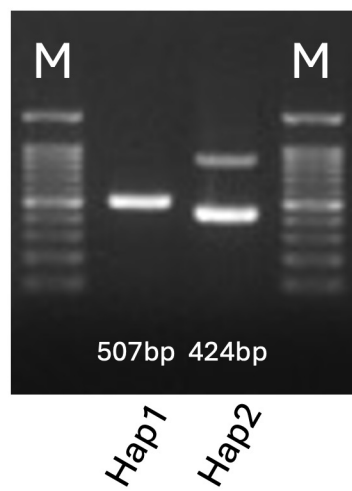

SV53

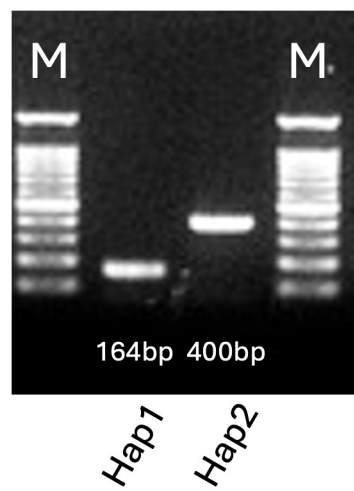

SV68

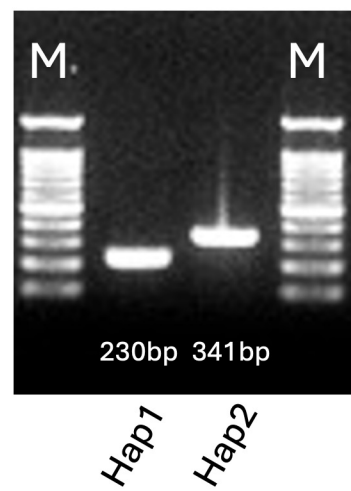

SV76

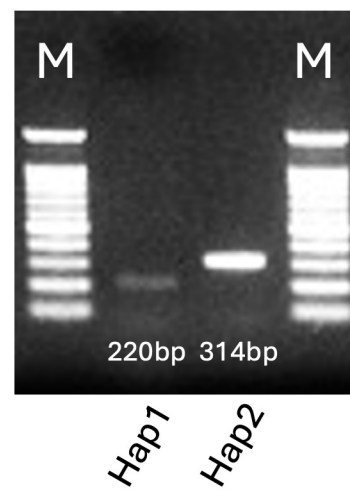

SV95

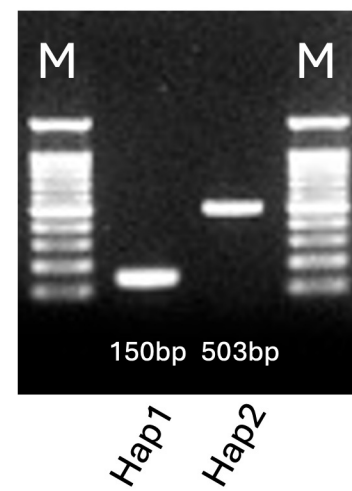

Supplement: Supplementary file 9 — Figure S8: Validation of SV markers. PCR products were resolved on a 2% agarose gel. PCR gel image of randomly selected five variants between hap‐1 and hap‐2 with ladder. [file PBI-24-4136-s001.pdf]

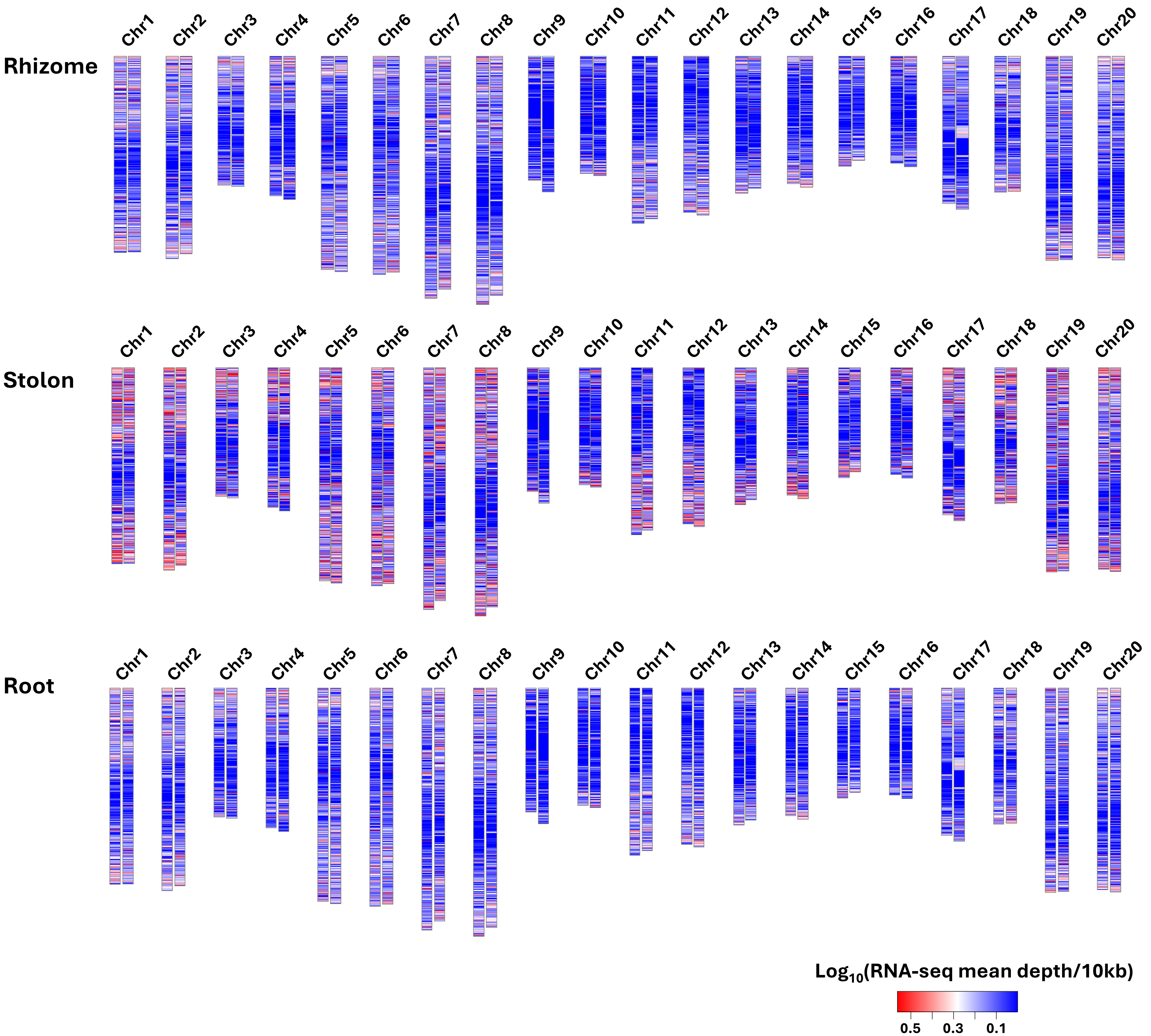

Supplement: Supplementary file 10 — Figure S9: Genome‐wide heatmap of RNA expression profiles in haplotype 1 and haplotype 2. From top to bottom, rows correspond to rhizome, stolon, and root. [file PBI-24-4136-s004.pdf]
